# Source-specific metabolic profiles and gene expression in areca nut cultivars from Hainan (China)

**DOI:** 10.3389/fpls.2025.1624083

**Published:** 2025-09-02

**Authors:** Yuanyuan Sun, Jian Feng, Haojie Liang, Shunan Zhang, Yuxia Wu, Anzhen Xie, Lin Zeng, Yangyang Liu

**Affiliations:** ^1^ Key Laboratory of Bioactive Substances and Resources Utilization of Chinese Herbal Medicine, Ministry of Education and National Engineering Laboratory for Breeding of Endangered Medicinal Materials, Institute of Medicinal Plant Development, Chinese Academy of Medical Sciences and Peking Union Medical College, Beijing, China; ^2^ Hainan Provincial Key Laboratory of Resources Conservation and Development of Southern Medicine and International Joint Research Center for Quality of Traditional Chinese Medicine, Hainan Branch of the Institute of Medicinal Plant Development, Chinese Academy of Medical Sciences and Peking Union Medical College, Haikou, China; ^3^ Hainan General Hospital, Hainan Affiliated Hospital of Hainan Medical University, Haikou, Hainan, China

**Keywords:** Areca nut, different sources, widely targeted metabolomics technology, transcriptome, alkaloids, flavonoids

## Abstract

*Areca catechu* L. are versatile plants whose fruits and seeds have great economic value. Although widely cultivated in Hainan Province, China, including native (HN) and introduced varieties from Taiwan (TW), Thailand (TG), and Vietnam (YN), the metabolic and regulatory differences among these varieties remain unclear. This study employed multi-omics techniques to analyze the metabolic profiles of phenylpropanoids, flavonoids, and alkaloids, along with their transcriptomic metabolic regulation, in the seeds of four areca nut germplasms. The research revealed significant metabolic differences among the seed varieties: the HN variety exhibited 175 differential metabolites, predominantly lignans, coumarins, and unsaturated fatty acids; the TW variety showed 270 differential metabolites with notably higher levels of flavonoids; the YN variety displayed 131 differential metabolites, characterized by intermediate overall metabolite levels but remarkably high glycerophospholipid content; and the TG variety contained 226 differential metabolites with relatively lower overall metabolite abundance. Furthermore, the key enzymes CCR and CHS were identified as core regulatory factors responsible for the differential accumulation of lignins, coumarins, and flavonoids observed between the HN and TW varieties. Overall, this study uncovers source-specific metabolic and regulatory distinctions in several AN varieties and provides further insights for enhancing AN utilization and supporting economic resilience for growers by identifying metabolites and biosynthetic pathways relevant to medicinal value and industrial processing.

## 1 Introduction

*Areca catechu* L., a member of the Palmae family, produces areca nuts (AN) that serve as a globally traded resource. Functioning both as significant traditional Chinese medicine for alleviating food stagnation and expelling parasites, and as a major chewing commodity ranking among the world’s top three oral stimulants, areca nuts are widely distributed across Indonesia, Philippines, Thailand, Vietnam, and China. According to FAO statistics, China (including Taiwan Province), Thailand, Vietnam, Indonesia, and India dominate recent international trade, with China accounting for approximately 5.26% of global imports (the highest share worldwide) and 21.59% of exports (Chen et al., 2025). Chinese regulatory authorities list areca nuts as non-first-time imported medicinal materials, primarily sourced from Southeast Asian countries like Thailand. Market surveys confirm that commercial medicinal areca nuts materials in China are overwhelmingly imported, with only minor contributions from Hainan province. Hainan itself cultivates over 118,600 hectares of *A. catechu* palms (Fu, 2020), making it China’s largest production region (Yang et al., 2018) and a vital economic crop—though primarily consumed as fresh young nuts or processed chewing products. Current Hainan plantings include both local varieties and introductions from Taiwan Province of China, Vietnam, and Thailand (Lu et al., 2018). Notably, foreign varieties face limited acceptance in chewing markets where Hainan types dominate, while Indonesian and Vietnamese cultivars are typically medicinal. Non-targeted metabolomics analysis by Zhang et al. (2023) revealed higher alkaloid content and superior medicinal quality in Hainan areca nuts compared to Indonesian and Thailand counterparts, confirming regional metabolic differences (Zhang et al., 2022); however, varietal distinctions and their associated bioactive compounds remain poorly defined, limiting medicinal/culinary optimization. Modern pharmacological studies demonstrate AN extracts possess antimicrobial, antiparasitic, anti-inflammatory, and antidepressant properties (Zhou et al., 2020; Jam et al., 2021; Serikuly et al., 2021; Puyathorn et al., 2022), alongside cardiovascular, gastrointestinal, neurological, and endocrine benefits (Dasgupta et al., 2018; Tey et al., 2021). Conversely, these extracts may promote oral diseases and carcinogenesis (Zhang et al., 2021; Xie et al., 2022; Liu and Chang, 2023). Currently, 88 compounds have been isolated and identified from areca nuts (AN), including alkaloids, flavonoids, phenolic acids, sterols, tannins, triterpenoids, and fatty acids. Among these, the main components that exert pharmacological effects are alkaloids and polyphenols (Sun et al., 2024). Alkaloids serve as diagnostic metabolites distinguishing Areca species from other palms (Peng et al., 2015). While the pharmacology of arecoline, arecaidine, guvacine, and guvacoline is well-studied, their biosynthetic pathways remain unclear. Current research emphasizes enhancing polyphenol extraction efficiency, investigating pharmacological activities, and elucidating flavonoid biosynthesis, given polyphenols’ high seed concentration and diverse benefits including antioxidant, anti-inflammatory, antimicrobial, and anticancer effects.

To further investigate the functions of metabolites in Areca catechu (abbreviated as AN), Lai et al. (2023) integrated metabolomics and transcriptomics to conduct a comparative analysis of the roots, stems, and leaves of *A. catechu*. This research identified and validated two UDP-glycosyltransferases and two novel transcription factors associated with the differential flavonoid content observed across these distinct plant parts. Moreover, while alkaloids, particularly pyridine-type alkaloids, have been extensively studied in areca nut research, the combined analysis of genomic and transcriptomic data has proven effective in revealing genes encoding trigonelline synthase (Li et al., 2022). Further, Zhou et al. utilized transcriptomics and targeted metabolomics to uncover transcription factors involved in regulating B-vitamins during areca nut development (Zhou et al., 2023). Wang et al. (2025), through haplotype-resolved genome sequencing coupled with integrated transcriptomic and metabolomic analysis, discovered that the AcGNMT1 and AcGNMT2 genes participate in the conversion of guvacine to arecoline, while AcUGTs function as glycosyltransferases; additionally, AcNMTs were identified as potential genes involved in arecoline biosynthesis (Zhou et al., 2025). Consequently, integrated multi-omics analysis has emerged as the prevailing approach for understanding environment-organism interactions and identifying key molecular mechanisms. Integrating metabolite accumulation profiles with gene expression data provides a more comprehensive understanding of biological system functionality.

Previous analyses by our research group on medicinal materials derived from different areca nut (AN) germplasms revealed notably higher arecoline content in Hainan varieties (up to 7.12 mg/g), compared to lower levels observed in germplasms from Taiwan (China), Vietnam, and Thailand (unpublished data). Therefore, this study constructs metabolite and transcriptome profiles for four AN varieties (originating from Hainan, China; Taiwan, China; Thailand; and Vietnam) to decipher their functional characteristics and biosynthetic pathways. This approach facilitates a deeper understanding of the metabolic traits specific to each AN variety, thereby supporting their optimal utilization.

## 2 Materials and methods

### 2.1 Plant materials

The experimental areca nut samples from four germplasms were collected from healthy, mature fruits harvested from trees over 7 years old cultivated in Dongcheng Town, Danzhou City, Hainan Province, China (19.44°N, 109.34°E). These included germplasms originally from Hainan, China (HN); Taiwan, China (TW); Thailand (TG); and Vietnam (YN). For each germplasm, fruits from three individual trees of the same variety were pooled together. All materials were flash-frozen in liquid nitrogen and subsequently stored at -80°C for subsequent analysis.

### 2.2 Metabolite extraction and metabolomic analysis

Each of the four areca nut types was sectioned to extract fresh seeds, which were then separately homogenized. Three replicate samples were subsequently collected from each germplasm’s homogenized seed material. Samples were freeze-dried in a vacuum freeze-dryer (Scientz-100F; Scientz, Ningbo, China) and then ground into powder using a grinder (MM 400; Retsch, Haan, Germany) at 30Hz for 1.5 minutes. Next, 50 mg of the powdered sample was combined with 1.2 mL of 70% methanol and vortexed for 30 seconds at 30 minute intervals for a total of six cycles. Samples were the centrifuged at 12,000 rpm for 3 minutes, with the obtained supernatants filtered through a 0.22 μm microporous membrane (SCAA-104, ANPEL, Shanghai, China) in preparation for UHPLC-MS/MS analysis. Three biological replicates were examined for each of the four group, and a quality control (QC) sample was generated by pooling 20 μL of supernatant from each individual sample.

For analysis, each sample was injected into a UPLC ExionLC™ AD system coupled with a QTRAP 4500 mass spectrometer (Sciex, Shanghai, China). Separation was performed using an Agilent Zorbax SB-C18 column (2.1mm × 100mm, 1.8 μm; Agilent Technologies, Santa Clara, California, USA) at a flow rate of 0.35 mL/min, an injection volume of 2 µL, and a temperature of 40°C. The mobile phase consisted of solvent A (0.1% formic acid in water) and solvent B (0.1% formic acid in acetonitrile) with the following elution gradient: a linear increase from 5%–95% solvent B from 0 to 9 minutes, maintained at 95% B for 1 minute, a linear decrease from 95%–5% B from 10 to 11 minutes, and maintained at 5% B for 3 minutes.

Mass spectrometry was performed with an electrospray ionization (ESI) source in both positive and negative modes with spray voltages of +5.5 kV/−4.5 kV, temperature at 550°C, ion source gas I at 50psi and gas II at 60psi, and curtain gas at 25psi. Collision-induced ionization was set to high energy and a mass-to-charge ratio (*m/z*) range from 100–1250 Da was employed. Multiple reaction monitoring (MRM) was performed using triple quadrupole (QQQ) and linear ion trap modes, with calibration and tuning performed using propylene glycol solutions at concentrations of 10 μmol/L and 100 μmol/L, respectively. QQQ scans were performed with the collision gas (nitrogen) at medium pressure. Total ion chromatogram (TIC) overlap for QC samples (**Supplementary Figures S1A, B**) and multi-peak detection patterns for MRM metabolites (**Supplementary Figures S1C, D**) were obtained.

Raw data was processed using Analyst (version 1.6.3, https://www.r-project.org). Metabolite data collection and identification was performed by comparing MS and MS/MS spectral data to Metware database (Metware Biotechnology Co., Ltd., Wuhan, China). The data was then exported to R software (version 1.0.1, www.r-project.org) for principal component analysis (PCA) and orthogonal partial least squares discriminant analysis (OPLS-DA). Differential metabolites were identified based on variable importance in the projection (VIP ≥ 1) and fold change (FC ≥ 2.0 or FC ≤ 0.5) values between groups.

### 2.3 RNA sequencing analysis

Total RNA was extracted from each seed sample of the four aforementioned areca nut germplasms using CTAB Reagent (Sangon Biotech Co., Shanghai, China) according to the manufacturer’s protocols.

RNA integrity was evaluated via gel electrophoresis, while RNA quantification and qualification were determined using an Invitrogen Qubit 4.0 Fluorimeter (Thermo Fisher Scientific, Massachusetts, USA), a SpectraMax M2 Multimode Microplate Reader (Molecular Devices, California, USA), and a Qsep400 bioanalyzer (Bioptic Inc., Taiwan, China). To isolate mRNA from the total RNA, oligo (dT) magnetic beads (Yeasen, Shanghai, China) were utilized and the obtained mRNA was then fragmented using a fragmentation buffer (Yeasen, Shanghai, China). Libraries were constructed using a Hieff NGS^®^ Ultima Dual-mode mRNA Library Prep Kit for Illumina^®^ (Yeasen, Shanghai, China) according to the manufacturer’s instructions (https://www.yeasen.com/products/detail/1780). Sequencing was performed using paired-end 150 bp reads on an Illumina NovaSeq 6000 platform (Illumina Inc., San Diego, California, USA) with three biological replicates prepared for each group.

To ensure a high sequence quality for further analysis, the raw sequencing data was preprocessed using fastp (version 0.23.2) to remove adapters and low-quality reads (Chen et al., 2018). Thus, the following reads were discarded: reads containing adapters, reads with over 10% “N” bases, and low-quality reads with more than 50% of the bases ≤ Q20. Clean reads were then mapped to the reference genome of *A. catechu* downloaded from NCBI using HISAT2 version 2.0.5 (https://ccb.jhu.edu/software/hisat2/index.shtml) (Kim et al., 2015).

### 2.4 Identification and analysis of differentially expressed genes

To determine corresponding gene expression levels, the FPKM (fragments per kilobase of transcript per million mapped reads) method was employed. Differential expression was determined using DESeq2 (version 1.20.0), with a statistical framework based on the negative binomial distribution used to analyze data from two experimental groups, each with three biological replicates (Love et al., 2014; Varet et al., 2016). To control the false discovery rate (FDR), *P*-values were adjusted using the Benjamini-Hochberg method. Genes were considered significantly differentially expressed if they met the threshold of FDR < 0.05 and |log_2_FC| > 1.

### 2.5 Metabolite and gene enrichment analysis

Identified metabolites were annotated using the KEGG database (http://www.kegg.jp/kegg/compound/) and subsequently mapped using the KEGG Pathway database (http://www.kegg.jp/kegg/pathway.html). Pathways containing significantly regulated metabolites were analyzed using Metabolite Set Enrichment Analysis, with significance determined based on the obtained hypergeometric *P*-values. Similarly, enrichment analysis for DEGs was conducted based on the hypergeometric test, employing pathway-based hypergeometric distribution for KEGG and GO term-based analysis for GO.

### 2.6 Quantitative real-time polymerase chain reaction

To confirm the RNA-seq findings, 7 DEGs were randomly selected and analyzed using qPCR. RNA extraction was performed using a RNAprep Pure Plant kit (DP441; Tiangen, Beijing, China) and first-strand cDNA synthesis was performed using a reverse transcriptase kit (KR118, Tiangen, Beijing, China). Samples were assessed using a EasyCycler 96 (Analytikjena, Jena, Germany) according to the manufacturer’s specified cycling conditions. Actin served as the internal control and relative expression levels were calculated with the 2^-ΔΔCT^ method. All primers utilized in this study are listed in **Supplementary Table S1** and samples were evaluated in triplicate.

## 3 Results

### 3.1 Overall metabolite analysis from different AN varieties

**Figure 1:**
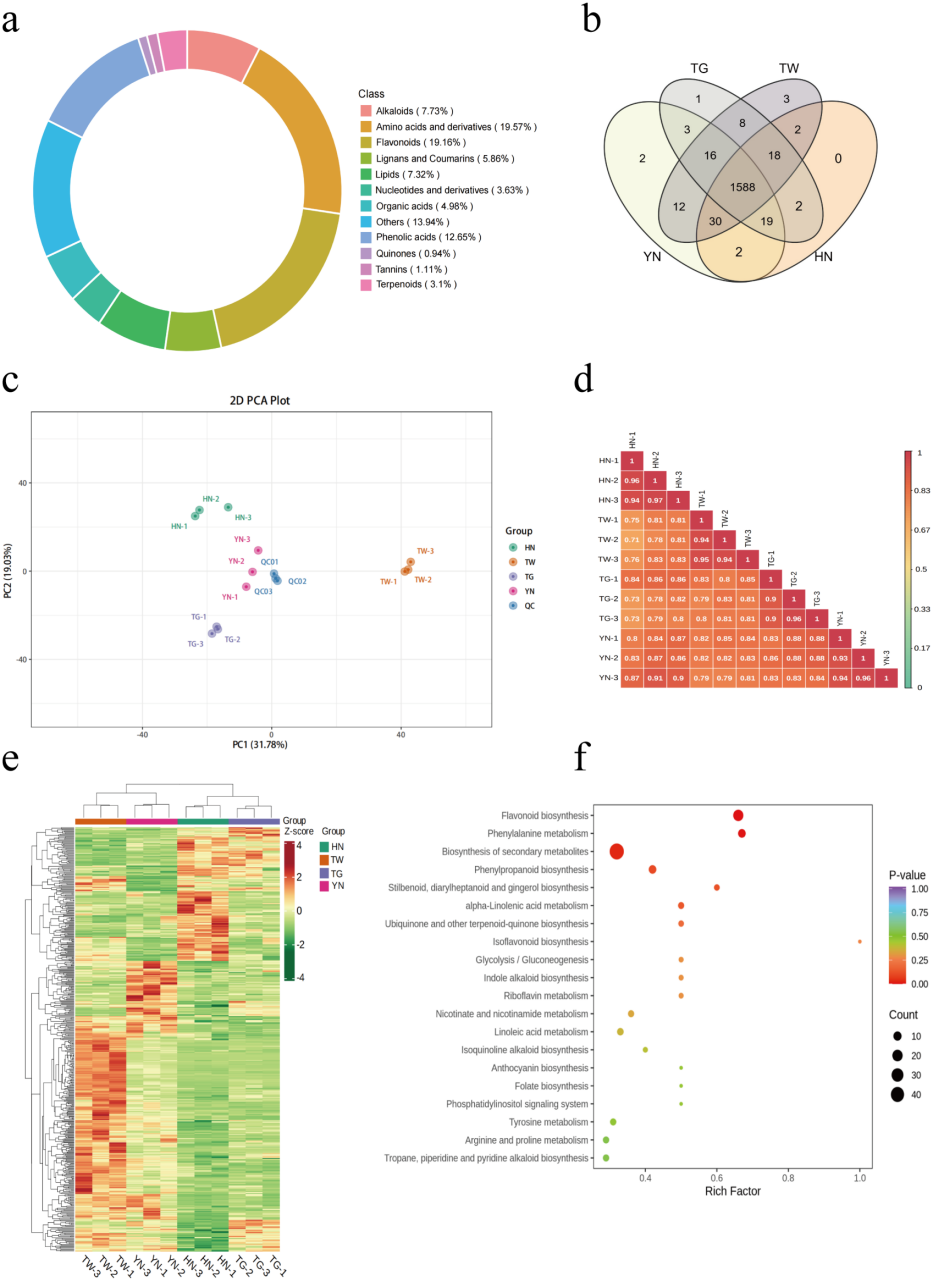
Metabolomic analysis of different AN varieties. **(a)** Ring diagram displaying the types and proportions of identified AN metabolites. **(b)** Venn diagram of metabolite numbers for the four groups. **(c)** PCA plots of the four groups. **(d)** Pearson correlation coefficients between all samples. **(e)** Cluster heatmap of all of the AN samples. **(f)** KEGG analysis of DAMs within the four groups.

Following UHPLC-MS/MS analysis, metabolites were identified base on Metware database, with 1,706 metabolites tentatively identified. Of these, 1,661 metabolites were detected within the HN group (*n =* 3), 1,672 metabolites in the YN group, 1,655 metabolites in the TG group, and 1,677 in the TW group (**Supplementary Table S2**). These metabolites were classified into 12 categories, including amino acids and derivatives (19.57%), flavonoids (19.16%), phenolic acids (12.65%), alkaloids (7.73%), lipids (7.32%), lignans and coumarins (5.86%), organic acids (4.98%), nucleotides and derivatives (3.63%), terpenoids (3.1%), tannins (1.11%), quinones (0.94%) and others (**Figure 1a**). Furthermore, while 1588 metabolites were shared by the four groups, several were unique to a given group, including the lipid component lysoPC 20:3 (TG only); the lipid component lysoPC 15:1 and alkaloid component N-caffeoylputrescine (YN only); and the alcohol component 2-amino-1,3-eicosanediol and flavonoids orientin-7-O-arabinoside and sakuranin (TW only; **Figure 1b**, **Table 1**).

**Table 1:** AN variety specific metabolites.

Group	Compounds	Class I	Class II
HN	–	–	–
TG	LysoPC 20:3	Lipids	Lysophosphatidylcholines
YN	LysoPC 15:1	Lipids	Lysophosphatidylcholines
	N-Caffeoylputrescine	Alkaloids	Phenolamine
TW	2-Amino-1,3-eicosanediol	Others	Alcohol compounds
	Orientin-7-O-arabinoside	Flavonoids	Flavones
	Sakuranin	Flavonoids	Flavanones

Differences between varieties and separation trends were analyzed using a PCA plot. The first two principal components explained 31.78% and 19.03% of the total variance, respectively (**Figure 1c**). The methods were confirmed to be reproducible and reliable due to closely clustered QC samples. Furthermore, all samples were unambiguously classified into five groups, implying a considerable variance in metabolite profiles between groups, but less within-group variation. Furthermore, correlation analysis showed the same strong correlation within a group and a lower correlation between groups (**Figure 1d**).

In this experiment, a randomized permutation test with 200 iterations revealed that the OPLS-DA model’s Q^2^ value for HN vs TW vs TG vs YN is 0.877 (*P* < 0.005; **Supplementary Figure S2**), indicating a stable and reliable model. To identify significantly differentially accumulated metabolites (DAMs) in the four groups, a criteria of VIP > 1.0 and *P* < 0.05 were employed, with 510 DAMs identified between the groups (**Supplementary Table S3**). Of these DAMs, the flavonoids, lipids, amino acids and derivatives, phenolic acids, and alkaloids accounted for a large proportion in all comparisons (**Figure 1e**). Additionally, the cluster heatmap of these DAMs shows that the TW sample had a higher content of DAMs, followed by the HN sample, and the TG sample had a lower content of DAMs. KEGG analysis was also employed to further examine the functional classifications of the identified DAMs from each group. The results identified “flavonoid biosynthesis,” “phenylalanine metabolism,” and “biosynthesis of secondary metabolites” as the most enriched pathways across the groups (**Figure 1f**).

### 3.2 DAMs between groups

**Figure 2:**
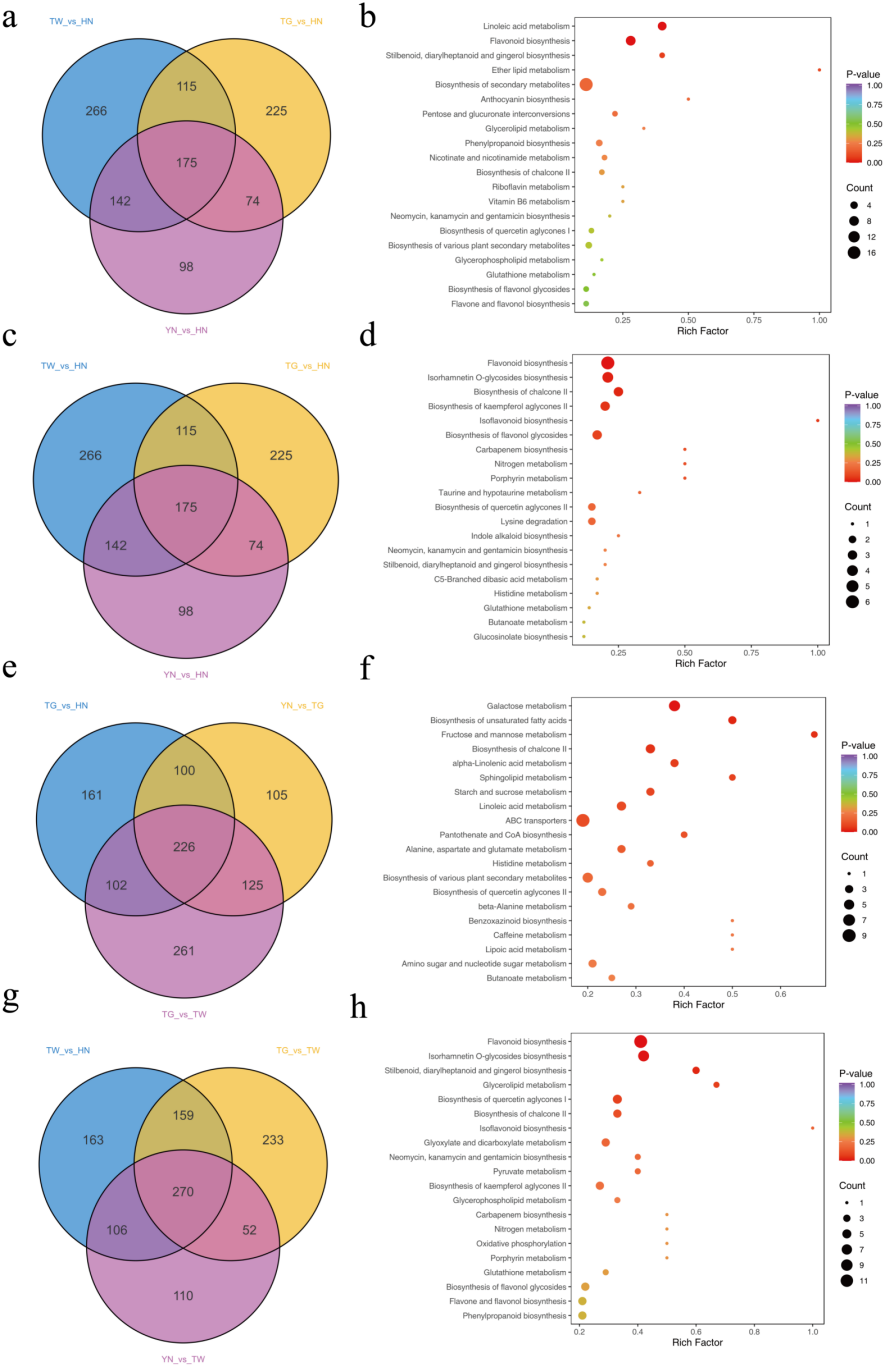
Analysis of common DAMs within a comparison group. Venn diagram of common DAMs when comparing HN vs YN, TG, and TW **(a)**, KEGG plot of common DAMs within comparison group HN vs YN, TG, and TW **(b)**, venn diagram of common DAMs when comparing YN vs HN, TG, and TW **(c)**, KEGG plot of common DAMs within comparison group YN vs HN, TG, and TW **(d)**, venn diagram of common DAMs when comparing TG vs YN, HN, and TW **(e)**, KEGG plot of common DAMs within comparison group TG vs YN, HN, and TW **(f)**, venn diagram of common DAMs when comparing TW vs HN, TG, and YN **(g)**, and KEGG plot of common DAMs within comparison group TW vs HN, TG, and YN **(h)**.

To further evaluate differences in metabolite profiles between groups, group comparisons were performed (**Supplementary Figure S3**). When comparing the TG and HN groups, 589 DAMs were identified (|log_2_FC| ≥ 1, VIP > 1), with 698 DAMs between TW and HN, and 489 DAMs between YN and HN. Notably, 175 DAMs were common to all three comparisons (HN vs. YN, TG, and TW; **Figure 2a**; **Supplementary Table S4**). Among these, 51 DAMs exhibited the highest accumulation in HN (FC ≥ 2), including pharmacologically active compounds such as 13(S)-HODE, 9,10-dihydroxy-12,13-epoxyoctadecanoic acid, ethyl linoleate, rabdosia acid A, α-hydroxylinoleic acid, piceid, resveratrol, pinoresinol, p-coumaryl alcohol, coniferyl alcohol, and caffeic acid. These metabolites are associated with anti-inflammatory, antimicrobial, antioxidant, and lipid-regulatory activities. KEGG enrichment analysis revealed “linoleic acid metabolism” and “flavonoid biosynthesis” as the most significantly enriched pathways (**Figure 2b**), both critical for plant stress resistance and bioactive compound synthesis.

When comparing the YN group to the TW, TG, and HN groups respectively, 131 common DAMs were identified (**Figure 2c**; **Supplementary Table S5**). Among these, 42 DAMs accumulated to the highest levels in the YN group (FC ≥ 2). Compared to the other groups, the pathways “Flavonoid biosynthesis,” “Isorhamnetin O-glycoside biosynthesis,” “Chalcone II biosynthesis,” and “Kaempferol aglycone II biosynthesis” were significantly enriched in the YN group (**Figure 2d**). For the TG group comparison with YN, TW, and HN varieties, 226 common DAMs were identified (**Figure 2e**; **Supplementary Table S6**). Of these, 41 metabolites accumulated most highly in TG (FC ≥ 2), while 146 accumulated at the lowest levels in TG (FC ≤ 0.5), indicating reduced metabolic activity in TG compared to other varieties. DAMs in the TG group were primarily enriched in the “Galactose metabolism,” “Unsaturated fatty acid biosynthesis,” “Fructose and mannose metabolism,” “Chalcone II biosynthesis,” and “α-Linolenic acid metabolism” pathways (**Figure 2f**), potentially associated with TG’s diminished stress resistance and reduced capacity for synthesizing bioactive compounds. In contrast, the TW group exhibited significant metabolic activity, with 270 common DAMs identified in its comparison (**Figure 2g**; **Supplementary Table S7**). Among these DAMs, the majority showed high accumulation levels (n = 214; FC ≥ 2), particularly phenolic compounds. These metabolites are associated with anti-fatigue, neuroprotective, cardioprotective, and anti-aging effects, likely mediated through alleviating oxidative stress and modulating inflammation. The enriched pathways aligned with previous comparative results, primarily emphasizing “Flavonoid biosynthesis” and “Isorhamnetin O-glycoside biosynthesis” (**Figure 2h**).

### 3.3 Transcriptome profiling via RNA-seq

Following RNA-seq, 98.37 Gb of raw data and 573,346,194 raw reads were obtained overall. Fast QC analysis showed that the Q30 scores of all samples ranged from 92.41% to 94.5% (**Supplementary Table S8**), indicating a high sequencing quality. After trimming adapter and low-quality reads, the remaining 539,668,076 reads were mapped to the *A. catechu* genome (http://arecaceae-gdb.com/#/download), with 91.69% successfully mapped to the reference genome (**Supplementary Table S9**).

To further evaluate the sequencing quality, PCA and Pearson’s correlation analysis were performed (**Supplementary Figures S4A, B**) and showed a high degree of correlation between HN and TG biological replicates (*r*=0.93−0.98), suggesting a high reproducibility. For the TW group, TW-2 showed a slightly lower correlation with TW-1 and TW-3 (*r*=0.86 and 0.71, respectively). YN showed a big difference in PC1 and a similarity in PC2 (*r*=0.86–0.90). The first two principal components explained 28.55% and 16.69% of the total variance, respectively.

To determine differential expression based on the RNA-seq data, DESeq2 was employed for samples with biological replicates, with |log_2_FC| ≥ 1 and FDR < 0.05 indicating differential expression. A total of 10,720 DEGs were identified in TG compared with HN, with 5,585 up-regulated and 5,135 down-regulated (**Supplementary Figure S5A**). In subsequent comparisons, TW vs. HN showed 2,512 up-regulated in TW and 2965 down-regulated; in YN vs. HN, 3,651 were up-regulated in YN and 2,063 down-regulated; in TG vs. TW, 5,491 were up-regulated in TG and 4,793 were down-regulated; in YN vs. TG, 3,083 were up-regulated in YN and 2,261 were down-regulated; and in YN vs. TW, 3,607 were up-regulated in YN and 1,630 were down-regulated.

**Figure 3:**
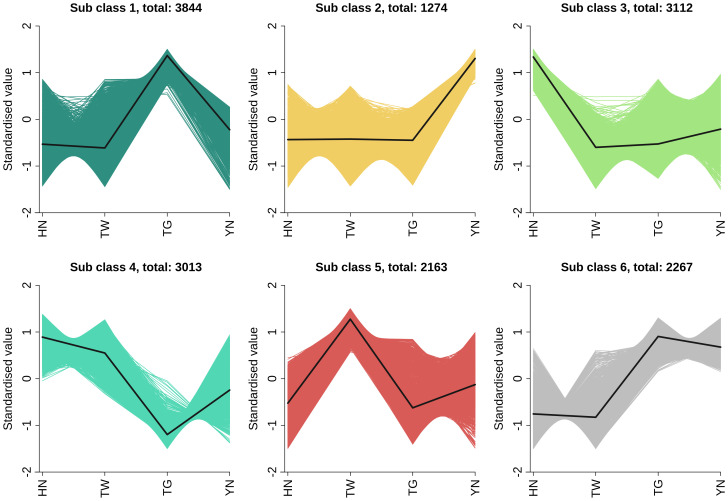
K-means cluster analysis of DAMs in HN vs. TW vs. TG vs. YN.

The union of DEGs across all comparisons resulted in a total of 15,676 DEGs in the overall sample. And then, K-means clustering analysis was performed on the DEGs identified in the overall sample. As shown in **Figure 3**, a total of 15,676 DEGs were classified into six categories. Class 1 (containing 3,844 DEGs) and class 6 (containing 2,267 DEGs) exhibited relatively higher expression primarily in the TG group; Class 2 (1,274 DEGs) showed enrichment mainly in the YN group; Class 3 (3,112 DEGs) and class 4 (3,013 DEGs) demonstrated predominant upregulation in the HN group; while class 5 (2,163 DEGs) displayed higher relative expression in the TW group. Overall, differentially expressed genes exhibited the highest expression levels in the HN and TG groups.

### 3.4 DEGs in different AN varieties

**Figure 4:**
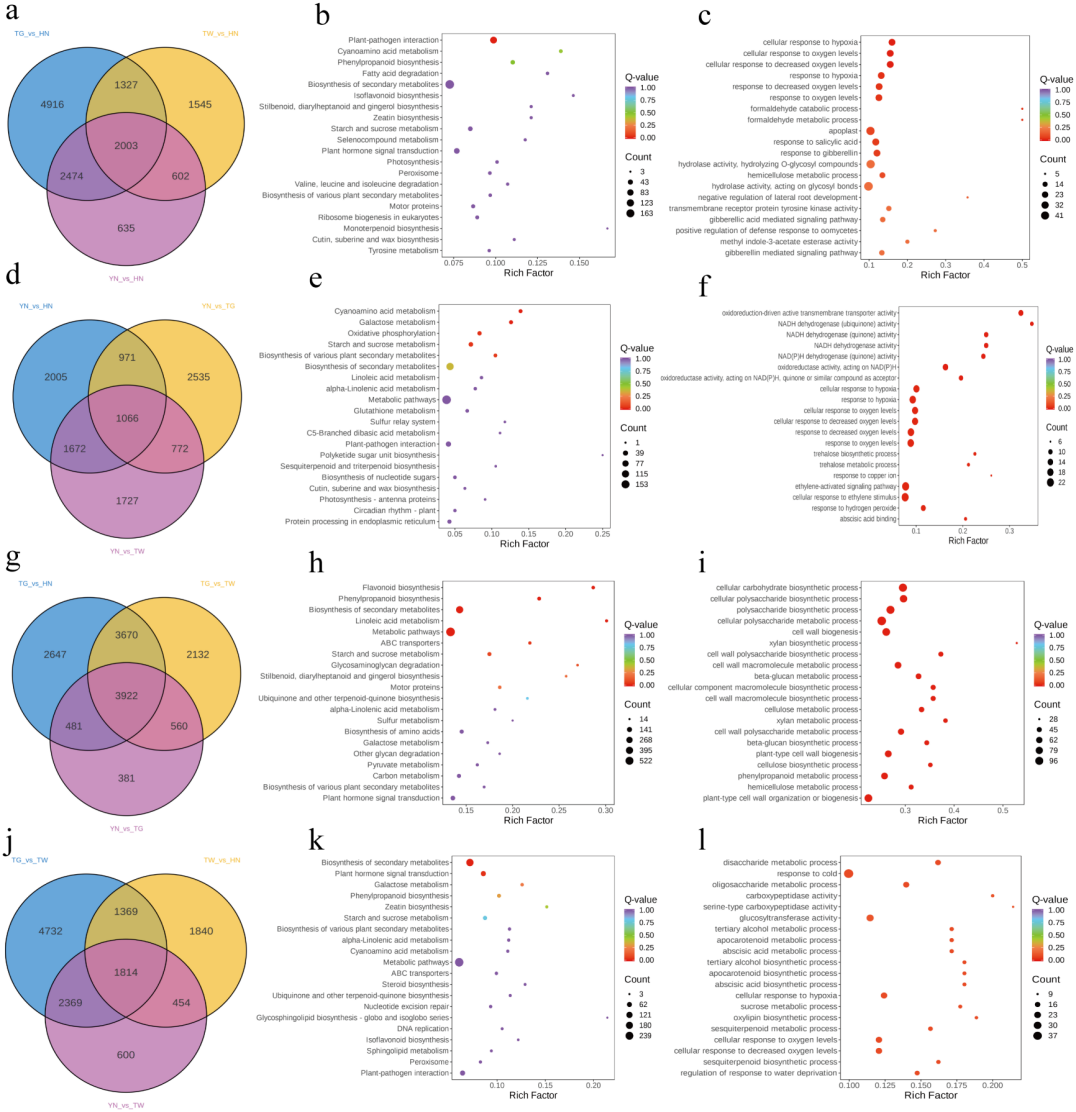
Analysis of common DEGs within a comparison group. Venn diagram of common DEGs when comparing HN vs YN, TG, and TW **(a)**, KEGG and GO plots of common DEGs within comparison group HN vs YN, TG, and TW **(b, c)**; venn diagram of common DEGs when comparing YN vs HN, TG, and TW **(d)**, KEGG and GO plots of common DEGs within comparison group YN vs HN, TG, and TW **(e, f)**; venn diagram of common DEGs when comparing TG vs YN, HN, and TW **(g)**, KEGG and GO plots of common DEGs within comparison group TG vs YN, HN, and TW **(h, i)**; venn diagram of common DEGs when comparing TW vs HN, TG, and YN **(j)**, KEGG and GO plots of common DEGs within comparison group TW vs HN, TG, and YN **(k, l)**.

To further evaluate associations, comparisons between HN and YN, TG, and TW were evaluated using a Venn diagram and 2,003 common DEGs (936 were up-regulated in HN and 876 were down-regulated; **Supplementary Table S10**) were identified (**Figure 4a**). KEGG pathway enrichment analysis revealed that these DEGs were significantly associated with pathways involved in organismal systems (“plant-pathogen interaction”), environmental information processing (“plant hormone signal transduction” and “MAPK signaling pathway-plant”), and metabolism (“starch and sucrose metabolism” and “phenylpropanoid biosynthesis”) (**Figure 4b**). Consistently, GO enrichment analysis in the biological process category highlighted terms related to plant defense and secondary metabolism, including “response to salicylic acid,” “response to oxygen/hypoxia,” and “phenol-containing compound metabolic/biosynthetic process” (**Figure 4c**). These findings align closely with the KEGG pathways of “plant-pathogen interaction” and “phenylpropanoid biosynthesis.” Additionally, in cellular component category, the DEGs were mainly enriched in the apoplast, microtubule cytoskeleton and cell wall, which locations commonly involved in defense responses to pathogenic attack. For molecular function, the enriched terms included hydrolase activity, transmembrane receptor protein kinase activity, and calcium ion binding, reflecting changes in metabolic and signaling processes.

Similarly, when comparing YN to each of the other groups, 1,066 common DEGs were noted (239 were up-regulated in YN and 47 were down-regulated; **Supplementary Table S11**), with the most enriched KEGG pathways including “cyanoamino acid metabolism”, “galactose metabolism”, “oxidative phosphorylation”, and “starch and sucrose metabolism” (**Figures 4d, e**). Corresponding GO terms were primarily related to defense and energy-related processes, such as “response to oxidative stress,” “disaccharide metabolic process,” and “oxidative phosphorylation” (**Figure 4f**), implicating roles in stress resistance, glucose metabolism, and mitochondrial function.

For TG, a total of 3,922 common DEGs were observed in the comparisons, with 1,783 genes upregulated and 2,032 downregulated in HN (**Supplementary Table S12**). These DEGs were mainly enriched in metabolic and secondary biosynthetic pathways, including “flavonoid biosynthesis,” “phenylpropanoid biosynthesis,” “biosynthesis of secondary metabolites,” “linoleic acid metabolism,” “metabolic pathways,” and “ABC transporters” (**Figures 4g, h**). GO enrichment highlighted biological processes such as “cellular carbohydrate biosynthetic process” and “cellular polysaccharide biosynthetic process” (**Figure 4i**). The predominance of downregulated DEGs in TG indicates a weaker transcriptional response related to stress resistance and secondary metabolism.

In comparisons involving TW, 1,814 common DEGs were identified, of which 724 were upregulated and 876 were downregulated in TW (**Supplementary Table S13**). These genes were significantly enriched in KEGG pathways such as “phenylpropanoid biosynthesis,” “biosynthesis of secondary metabolites,” and “plant hormone signal transduction” (**Figures 4j, k**). GO analysis further indicated enrichment in categories related to abiotic stress response, secondary metabolite biosynthesis, and hydrolase activity (**Figure 4l**), suggesting transcriptional modulation of key defense and metabolic processes in TW.

### 3.5 Integrated analysis of DEGs and DAMs

To investigate potential associations between common DEGs and DAMs, an integrated analysis was performed. To control the false positive risk associated with multiple testing, enhance statistical power, focus on metabolites most likely possessing core biological significance, and ensure manageable visualization and interpretation of results, the top 50 metabolites showing the strongest associations with differentially expressed genes (DEGs) and differentially accumulated metabolites (DAMs) were selected for correlation analysis. Pearson correlation coefficients (PCCs) between the top 50 DEGs and DAMs were calculated using the Cor package in R, with PCCs > 0.8 and *P* < 0.05 deemed significant. The results revealed significant correlations in HN (*n =* 201), TW (*n =* 744), TG (*n =* 83), and YN (*n =* 288; **Supplementary Table S14**), with both positive and negative correlations observed (**Supplementary Figure S6**).

Additionally, the DEGs and DAMs from the four comparisons were co-mapped based on the KEGG database to isolate the top 50 common pathways (**Supplementary Figure S7**). In the TG comparison group, co-enrichment pathways were noted, with “galactose metabolism” significantly enriched and “flavonoid biosynthesis,” “phenylpropanoid biosynthesis,” “linoleic acid metabolism,” “metabolic pathways,” and “starch and sucrose metabolism” showing a higher degree of co-enrichment. For the TW comparison group, co-enrichment pathways were identified, including “flavonoid biosynthesis” and “biosynthesis of secondary metabolites.” The HN comparison group showed co-enrichment pathways, including “cyanoamino acid metabolism,” “phenylpropanoid biosynthesis,” “flavonoid biosynthesis,” and “linoleic acid metabolism;” while 26 co-enriched pathways were identified in the YN comparison group, including “biosynthesis of secondary metabolites,” “metabolic pathways,” “glutathione metabolism,” and “flavonoid biosynthesis.”.

#### 3.5.1 Distribution of DAMs and DEGs of different AN in phenylpropanoid and flavonoid biosynthesis

**Figure 5:**
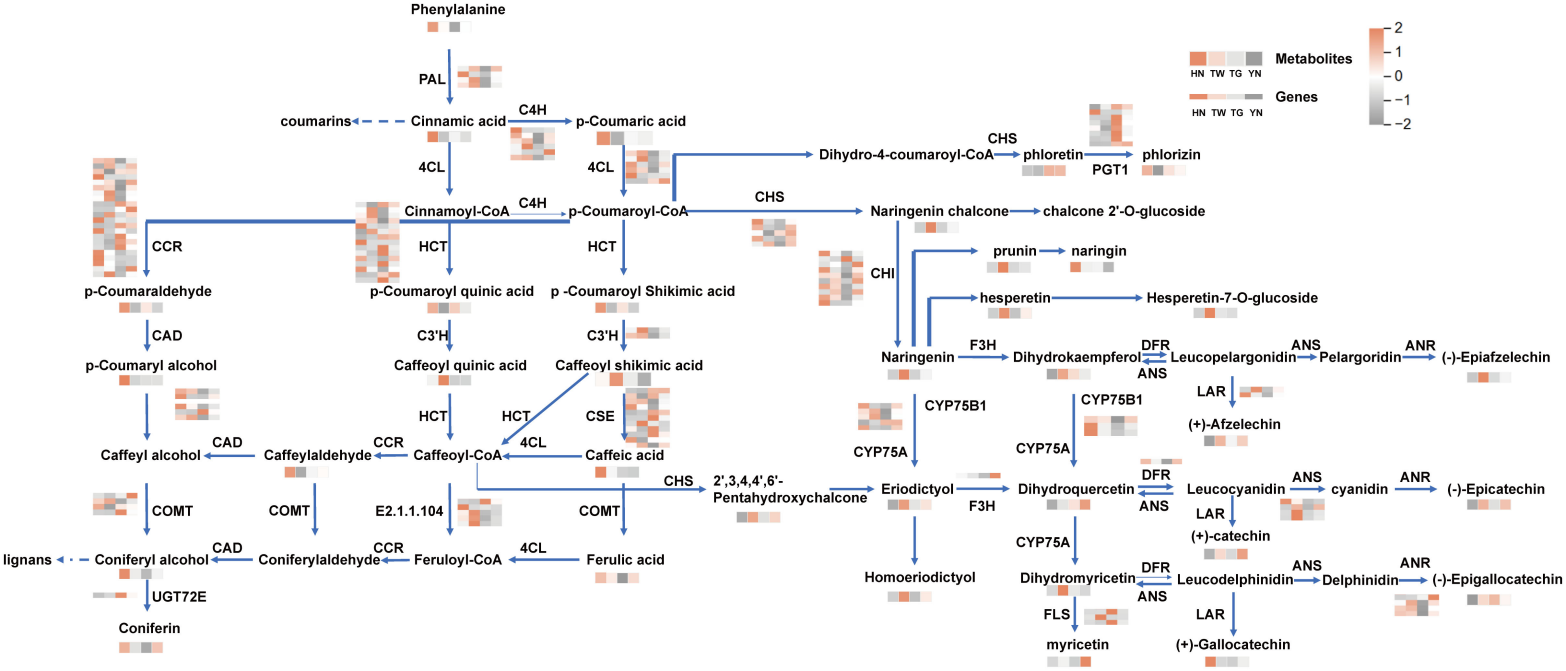
Differences in metabolite content and gene expression in phenylpropanoid and flavonoid biosynthesis pathways of areca nut from four different sources: HN, TW, TG, YN. The darker the brown, the higher the content of the corresponding metabolites or the higher the gene expression, and the darker the gray, the lower the content of the corresponding metabolites or the lower the gene expression.

Herein, phenylpropanoid biosynthesis and flavonoid biosynthesis were the two KEGG pathways that were predominantly enriched following DAM and DEG co-enrichment analysis. In the phenylpropanoid pathway, L-phenylalanine is converted into cinnamic acid by phenylalanine ammonia-lyase (PAL), with cinnamic acid then converted into a coumarin precursor via cinnamic acid 4-hydroxylase (C4H). Thus, biosynthesis and regulatory pathways associated with flavonoids and phenylpropanoids were mapped in KEGG (**Figure 5**), and 118 DEGs were identified, including *PAL*, *4CL*, *C4H*, *CCR*, *CSE*, *CAD*, *COMT*, *CHS*, *CHI*, *F3H*, *DFR*, *ANS*, *ANR*, *LAR*, *HCT*, *C3′H*, and *FLS*. The HN group showed the highest level of coumarin analogues, including L-phenylalanine and cinnamic acid. Furthermore, the HN group showed the highest levels of coniferyl alcohol, a key precursor in lignin synthesis. Moreover, other components associated with the formation of coniferyl alcohol were also noted in the HN group, including p-coumaraldehyde, p-coumaroyl quinic acid and p-coumaroyl shikimic acid and genes *HCT* (*Acat_11g001720*, *Acat_6g006370*, *Acat_9g024310*), *CAD* (*Acat_10g014850*, *Acat_11g019720*), *CSE* (*Acat_14g000490*), and *CCR* (*Acat_10g019380*, *Acat_8g011290*). In relation to the flavonoid synthesis pathway, p-coumaric acid was higher in HN, while other pathway components, including naringenin chalcone, naringenin, prunin, hesperetin, hesperetin-7-O-glucoside, eriodictyol, 2’,3,4,4’,6’-pentahydroxychalcone, epiafzelechin, dihydrokaempferol, and dihydromyricetin, showed a higher accumulation in TW. Curiously, however, some of the genes that encode enzymes associated with this pathway were not more highly expressed in TW, but were highly expressed in HN, to include *CHI*, *CYP75B1*, *F3H* and *DFR*. This is quite consistent with the expected judgment results. Additionally, some of the downstream products, afzelechin, phlorizin, myricetin, gallocatechin and epigallocatechin were not the highest in TW. Therefore, these results suggest that while the levels of the synthesized upstream metabolites were not the highest in TW, the low downstream consumption may have led to the accumulation of flavonoid substances and thus such substances are more abundant in TW relative to HN. Therefore, the accumulation and changes of key substances in the biosynthesis of phenylpropanoids and flavonoids contributed to the differences among areca nut germplasms.

#### 3.5.2 Distribution of DAMs and DEGs of different AN in alkaloids biosynthesis

**Figure 6:**
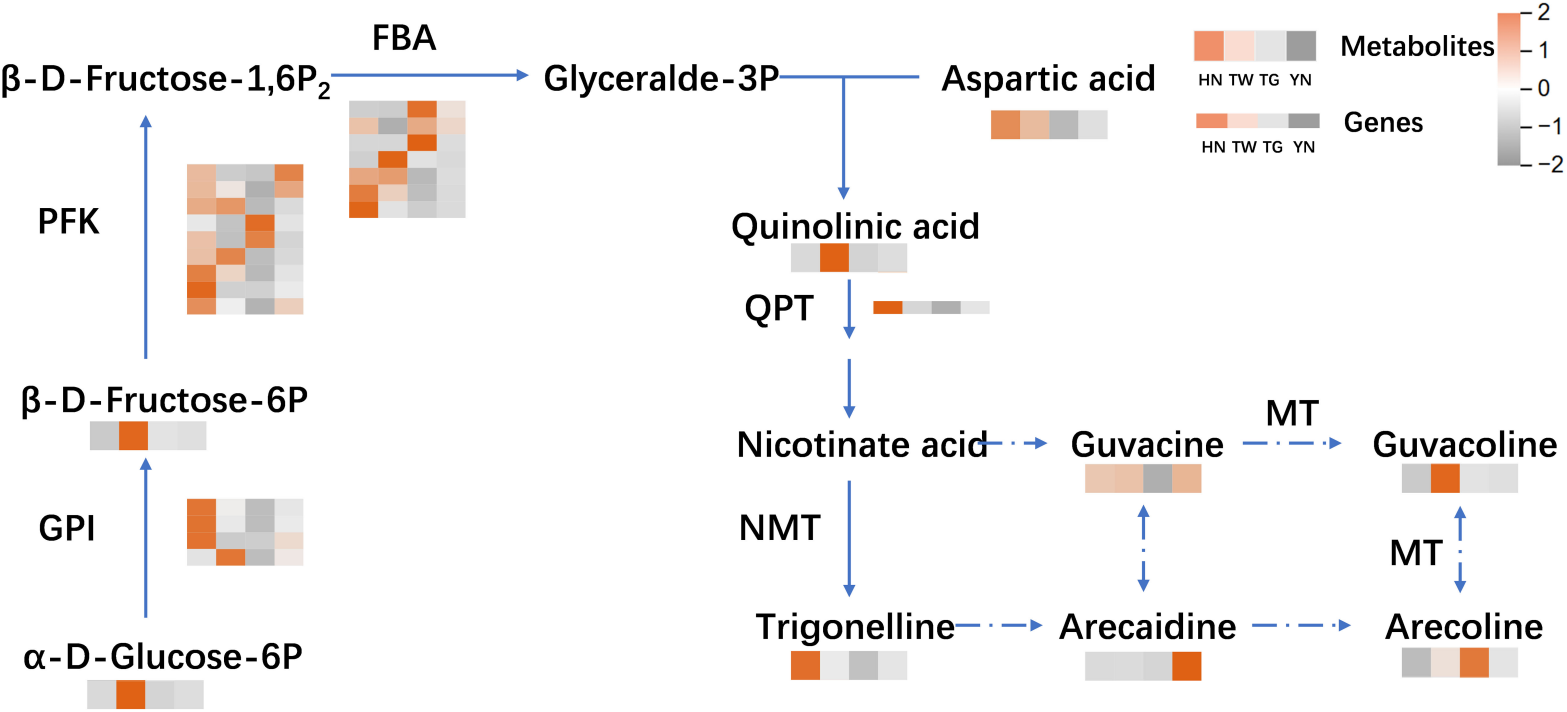
The biosynthesis pathway of arecine in areca nut and the differences in metabolite content and gene expression in arecine synthesis pathway of areca nut from four different sources: HN, TW, TG, YN. The darker the brown, the higher the content of the corresponding metabolites or the higher the gene expression, and the darker the gray, the lower the content of the corresponding metabolites or the lower the gene expression.

The pyridine alkaloids, which are represented by arecoline, arecaidine, guvacine, and guvacoline, are the main types of AN alkaloids. In a previous study examining AN biosynthesis pathway, the biosynthesis of arecoline was hypothesized to be complemented by the enzyme-catalyzed formation of trigonelline and 3,6-dihydronicotinic acid from nicotinic acid. These were then both used in the actions of reductase and isomerase enzymes to produce guvacine and guvacoline, respectively, and then methyltransferase enzymes produced arecoline and arecaidine (Xu et al., 2021). Based on this hypothesis, as well as the metabolites and genes actually detected in this experiment, the relevant pathways from glucose metabolism to alkaloid synthesis are briefly mapped. As is shown in the **Figure 6**, Trigonelline and quinolinic acid are key metabolites in the arecoline synthesis. Quinolinic acid was more abundant in TW samples than in other groups, probably due to the high accumulation of metabolites such as α-D-glucose-6P in TW, even though the expression of genes for the related enzymes was higher in HN. Trigonelline was higher in HN samples than in other groups, probably due to the high expression of genes related to QPT enzymes in HN. However, arecoline, arecaidine, guvacine, and guvacoline did not have a clear distribution pattern in the four groups of samples.

### 3.6 Transcriptome validation via qPCR

To validate the accuracy of the transcriptomic data, 3 DEGs (*4CL*, *Acat_13g005110*; *C4H*, *Acat_14g019800*; and *CAD*, *Acat_10g014850*) related to phenylpropanoid biosynthesis, and 4 DEGs (*F3H*, *Acat_3g013610*; *CYP75A*, *Acat_1g003840*; *ANS*, *Acat_9g011730*; and *LAR*, *Acat_14g006550*) associated with flavonoid biosynthesis were selected for verification using qPCR. Similar expression profiles were observed when comparing the qPCR and RNA-Seq findings (**Supplementary Figure S8**), suggesting that the transcriptomic results are reliable.

## 4 Discussion

### 4.1 The differences in HN, YN, TG, and TW

When comparing HN to each of the other groups, YN, TG and TW, 175 common DAMs were identified and 2,003 common DEGs were identified. Unsaturated fatty acids, a key defense system against biotic and abiotic stresses (He and Ding, 2020), were significantly enriched in HN. The elevated lipid content (≥2-fold vs other groups) and upregulation of DEGs in the plant-pathogen interaction pathway indicate HN’s robust stress-responsive capacity. Furthermore, HN exhibited the highest lignan and coumarin levels, which correlated with the overexpression of DEGs in phenylpropanoid biosynthesis. These metabolites likely underpin HN’s pharmacological properties, including anti-inflammatory, antibacterial, and lipid metabolism-regulatory activities.

In contrast, YN shared 131 common DAMs and 1,066 common DEGs across comparisons. YN displayed the lowest number of extreme DEGs (YN: 239-up and 47-down; HN: 936-up and 876-down; TG: 1783-up and 2032-down; TW: 724-up and 876-down) and intermediate metabolite levels. However, its enrichment of DEGs in energy metabolism and glucose metabolism pathways corresponded to elevated polyphenol and alkaloid content, which may contribute to cardiovascular and neuroprotective effects.

For TG, 589 common DAMs and 3,922 common DEGs were identified. Notably, 51.8% of DEGs (*n =* 2,032) in key pathways were downregulated, including flavonoid biosynthesis, phenylpropanoid biosynthesis, and linoleic acid metabolism. This transcriptional suppression coincided with reduced metabolite levels in critical pathways: galactose metabolism, unsaturated fatty acid biosynthesis, and chalcone II biosynthesis, leading to the continued impairment of stress resistance. TW showed 270 common DAMs and 1,814 common DEGs. Unlike TG, 79.3% of DAMs (*n =* 214) were upregulated, particularly phenolics (78 flavonoids and 40 phenolic acids). Key metabolites included quercetin and chlorogenic acid, which are linked to anti-fatigue, cardioprotective, and neuroprotective effects. However, TW’s downregulation of DEGs in plant hormone signal transduction and plant-pathogen interaction, coupled with low lipid, organic acid, lignan, and coumarin levels, suggests compromised systemic stress resilience despite localized phenolic accumulation.

### 4.2 The biosynthesis of phenylpropanoid and flavonoid

In plants, secondary metabolites are not only important to the plant itself, but many have important medicinal and industrial value. There are a variety of secondary metabolites in AN, with polyphenols being one of the important metabolites. Phenolic substances are divided into flavonoid polyphenols, including isoflavones, flavanones, flavanols, anthocyanins, flavonones, and flavonols, and non-flavonoid polyphenols, including lignans, phenolic acids, stilbens, xanthones, and tannins (Durazzo et al., 2019). Flavonoids are by far the largest group of polyphenols with medicinal antioxidant, antimicrobial, and anti-inflammatory activities. While previous studies have also utilized a combined multi-omic approach to examine flavonoid biosynthesis pathways in AN (Lai et al., 2023), this study provides a more detailed pathway map by combining phenylpropanoid biosynthesis and flavonoid biosynthesis. Herein, lignans and coumarins, other important phenolic compounds, were highlighted, with these compounds considered plant estrogens and found to have medicinal antibacterial, liver protective, and antioxidant activities. Coumarins are phytoalexins that can rapidly accumulate when plants are attacked by pathogens or under abiotic stresses (Venkata Sairam et al., 2016). While the biosynthetic pathways associated with lignans and coumarins have not yet been fully elucidated, it is now widely believed that coniferyl alcohol is the precursor of lignin synthesis (Liu et al., 2016) and cinnamic acid is the starting component of coumarin-like compounds (Wang et al., 2022). Furthermore, all of these components, flavonoids, lignans, and coumarins, originate from phenylalanine. In this study, HN samples possessed higher phenylalanine, cinnamic acid and pinocyanol and also possessed higher lignan and coumarin content, including hedyotol C, pinoresinol, epipinoresinol, trachelogenin, 3,4-dihydrocoumari, and fraxetin-7,8-di-O- glucoside. TW samples had higher flavonoid content and a more active flavonoid biosynthesis pathway, with higher hesperidin, naringenin, eriodictyol, homoeriodictyol, and dihydromyricetin content. Studies have reported that the transcription factors AcMYBs involved in flavonoid biosynthesis in areca nut (Lai et al., 2023; Jiang et al., 2025), while chalcone synthase and chalcone isomerase ACCHs can regulate the accumulation of flavonols (Yu et al., 2023). Metabolite accumulation and gene regulation were found to be closely associated when examining chalcone synthase (CHS) and chalcone reductase (CCR), important enzymes in phenylpropanoid biosynthesis. CHS is the rate-limiting enzyme in flavonoid biosynthesis (Dao et al., 2011), and CCR is the key enzyme for the entry into the lignin-specific pathway from the phenylpropanoid pathway in plants and plays an important role in the regulation of lignin biosynthesis (Fan et al., 2015). In this study, CHS appears to affect flavonoid biosynthesis by catalyzing the formation of naringenin chalcone and 2’,3,4,4’,6’-pentahydroxychalcone using p-coumaroyl-CoA and caffeoyl-CoA as substrates, respectively. Furthermore, it would appear that CCR uses p-coumaroyl-CoA and caffeoyl-CoA as substrates, catalyzing the formation of p-coumaraldehyde and caffeylaldehyde, respectively. These products then appear to function as substrates for subsequent reactions associated with phenylpropanoid biosynthesis. Moreover, previous studies have shown that CHS competes with CCR for p-coumaroyl-CoA and caffeoyl-CoA binding, thus resulting in differences in flavonoid biosynthesis and phenylpropanoid biosynthesis activity. In this study, the TW groups showed an increased expression in CHS-related gene (Acat_9g019130), which was accompanied by a higher accumulation of flavonoid biosynthesis related metabolites. Conversely, in the HN group, the increased expression of CCR-related genes (Acat_10g019380 and Acat_8g011290) was correlated with a higher accumulation of phenylpropanoid biosynthesis and lignin biosynthesis metabolites.

### 4.3 The biosynthesis of arecoline

Thus far, the biosynthetic pathways of alkaloids in AN have not been fully resolved, and the prevailing view remains that pyridine alkaloids are formed from nicotinic acid, nicotinic acid is generated from quinolinic acid, and quinolinic acid is derived from L-aspartic acid with 3-phosphoglyceraldehyde (Katoh et al., 2006; Ashihara et al., 2015). Herein, the source of piperidine alkaloid formation, L-aspartic acid, was found at a higher concentration in HN, while its downstream product, quinolinic acid, showed higher levels in TW, so the key may lie in 3-phosphoglyceraldehyde. However, 3-phosphoglyceraldehyde, which is produced in the glycolytic pathway, was not detected during this metabolomic analysis. Upon examining relevant pathways, metabolites associated with starch and sucrose metabolism, versus galactose metabolism, were more active in TW; but HN had higher relevant gene expression. Thus, we postulate that more sugars were consumed in the HN group for resistance to external stimuli, resulting in less synthesized 3-phosphoglyceraldehyde, whereas TW accumulated more glucose and fructose, and thus may produce more 3-phosphoglyceraldehyde.

However, unfortunately, associated components, including nicotinic acid, trigonelline, quinolinic acid, arecoline, arecaidine, guvacine, and guvacoline, did not show a clear distribution pattern among the four groups examined, due to the lack of specificity in the broad-targeted metabolomics approach. Moreover, nicotinate N-methyltransferase and quinolinic acid phosphoribosyltransferase were not detected in this experiment. Thus, this experiment did not provide further insights into the biosynthetic pathway of arecoline, which will require targeted detection and analysis of alkaloids in the future.

## 5 Conclusion

In this study, four AN varieties were examined using both metabolomic and transcriptomic assays. When comparing each group to every other group to isolate common differences, common DAMs within each comparison, including HN (*n =* 175), TW (*n =* 270), TG (*n =* 226), and YN (*n =* 131), were identified. The HN group showed higher levels of lignans and coumarins, unsaturated fatty acids, and organic acids, such as hedyotol C, epipinoresinol, pinoresinol, γ-linolenate, 9(S)-HPODE, 13(S)-HPODE, 9,10-dihydroxy-12,13-epoxyoctadecanoate, and 2-methylglutaric acid; while the TW group displayed higher flavonoid accumulation, including naringenin, dihydrokaempferol, epicatechin, and epiafzelechin. YN and TG groups showed mostly moderate metabolite accumulation levels, but with high levels of glycerophospholipids, LysoPC 15:1 and LysoPE 18:1, noted in YN. Co-enrichment analysis of the identified DAMs and DEGs showed that phenylpropane and flavonoid biosynthesis were the most significantly enriched pathways. Specifically, HN samples had higher metabolite and gene expression associated with phenylpropanoid biosynthesis, while TW samples had higher metabolite and gene expression associated with flavonoid biosynthesis. Additionally, linoleic acid and α-linolenic acid metabolism, as well as starch, sucrose, and galactose metabolism were also significantly enriched pathways that are associated with plant stress responses and energy availability. While the findings presented herein have aided in further elucidating biosynthetic differences between different AN varieties, further comprehensive testing is necessary to more fully characterize the noted differences and any possible associated advantages. Additionally, these profiles may aid in identifying genes affecting the synthesis of key metabolites to improve AN variety selection, as well as to enable the targeting of certain metabolite content.

## Data Availability

The datasets presented in this study can be found in online repositories. The names of the repository/repositories and accession number(s) can be found in the article/**Supplementary Material**.
