# Supplementary material for: Source-specific metabolic profiles and gene expression in areca nut cultivars from Hainan (China)
Source: Front Plant Sci. 2025 Sep 2;16:1624083. doi: 10.3389/fpls.2025.1624083 (PMC12436652; doi:10.3389/fpls.2025.1624083)
Supplement: Supplementary file 2 [file DataSheet2.docx]

Supplementary Material


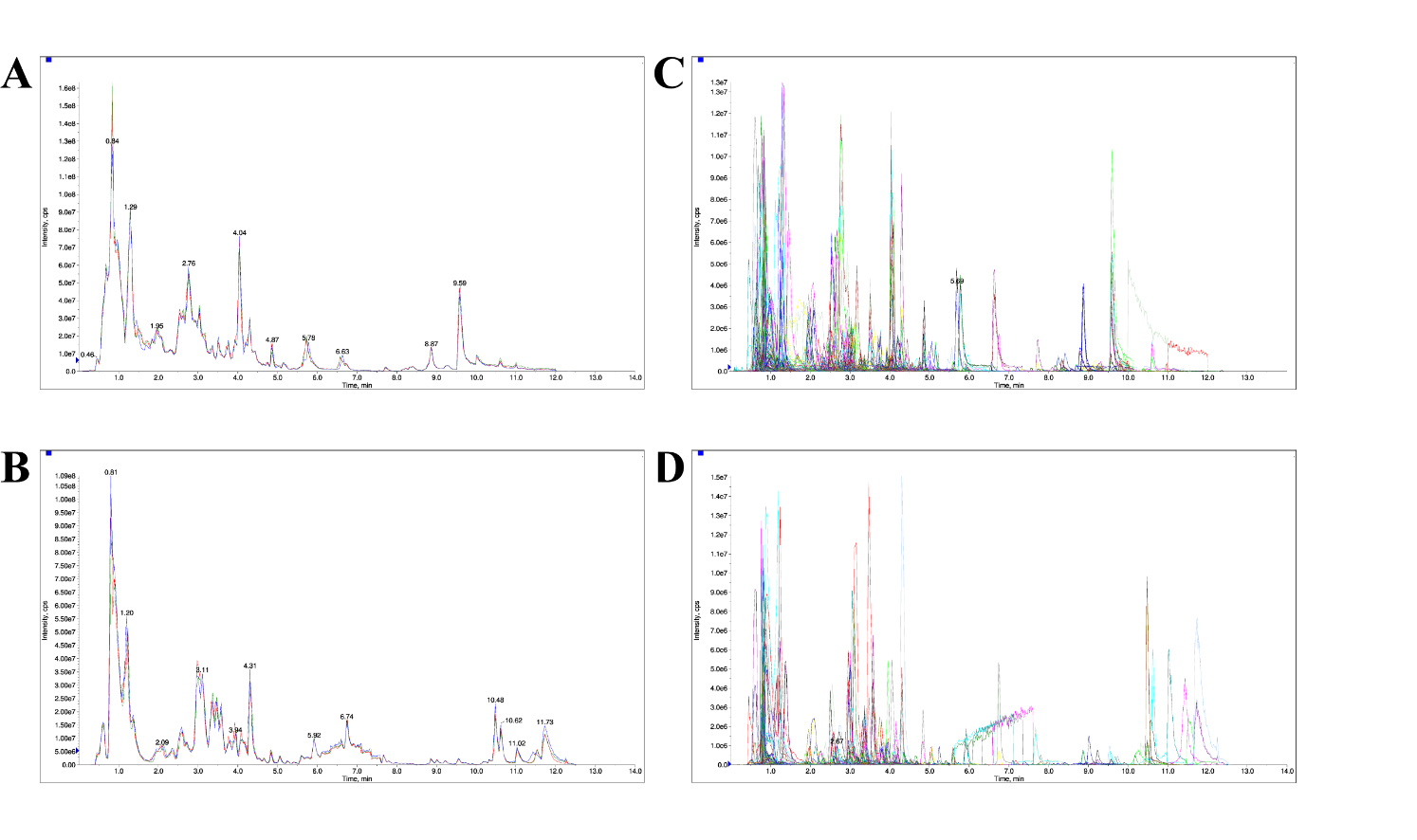


**Supplementary Figure 1.** The total ion chromatogram (TIC) overlap diagram of the QC samples from positive (A) and negative (B) ESI modes. The multi-peak pattern of MRM metabolite from positive (C) and negative (D) ESI modes.


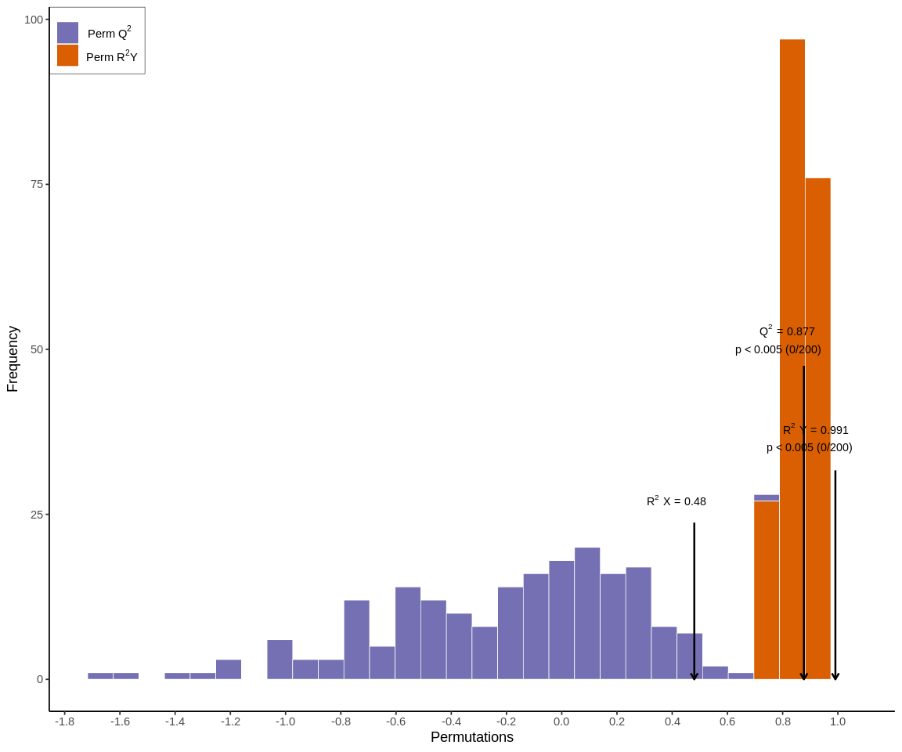


**Supplementary Figure 2.** Validation plot for the OPLS-DA model.


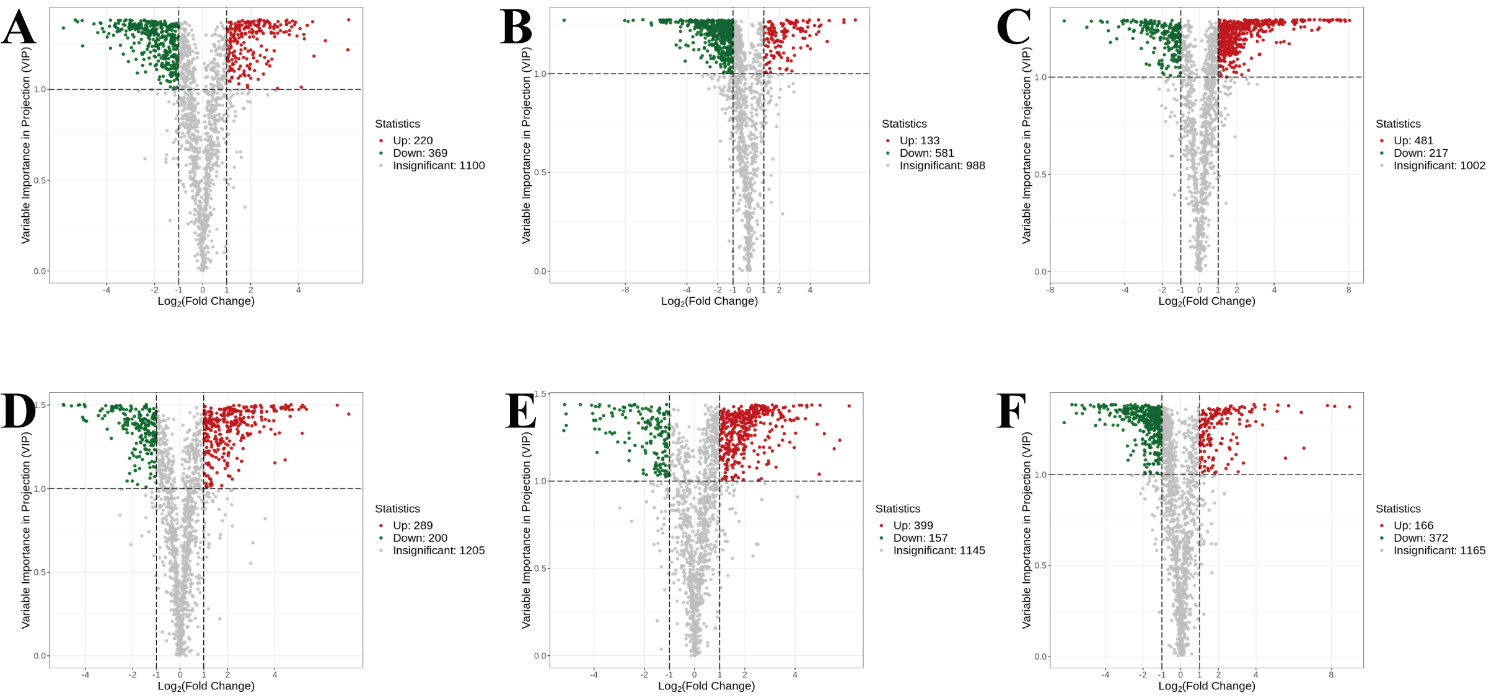


**Supplementary Figure 3.** Volcanic maps of DAMs in different contrasts. (A) TG vs HN; (B) TG vs TW; (C) TW vs HN; (D) YN vs HN; (E) YN vs TG; (F) YN vs TW.


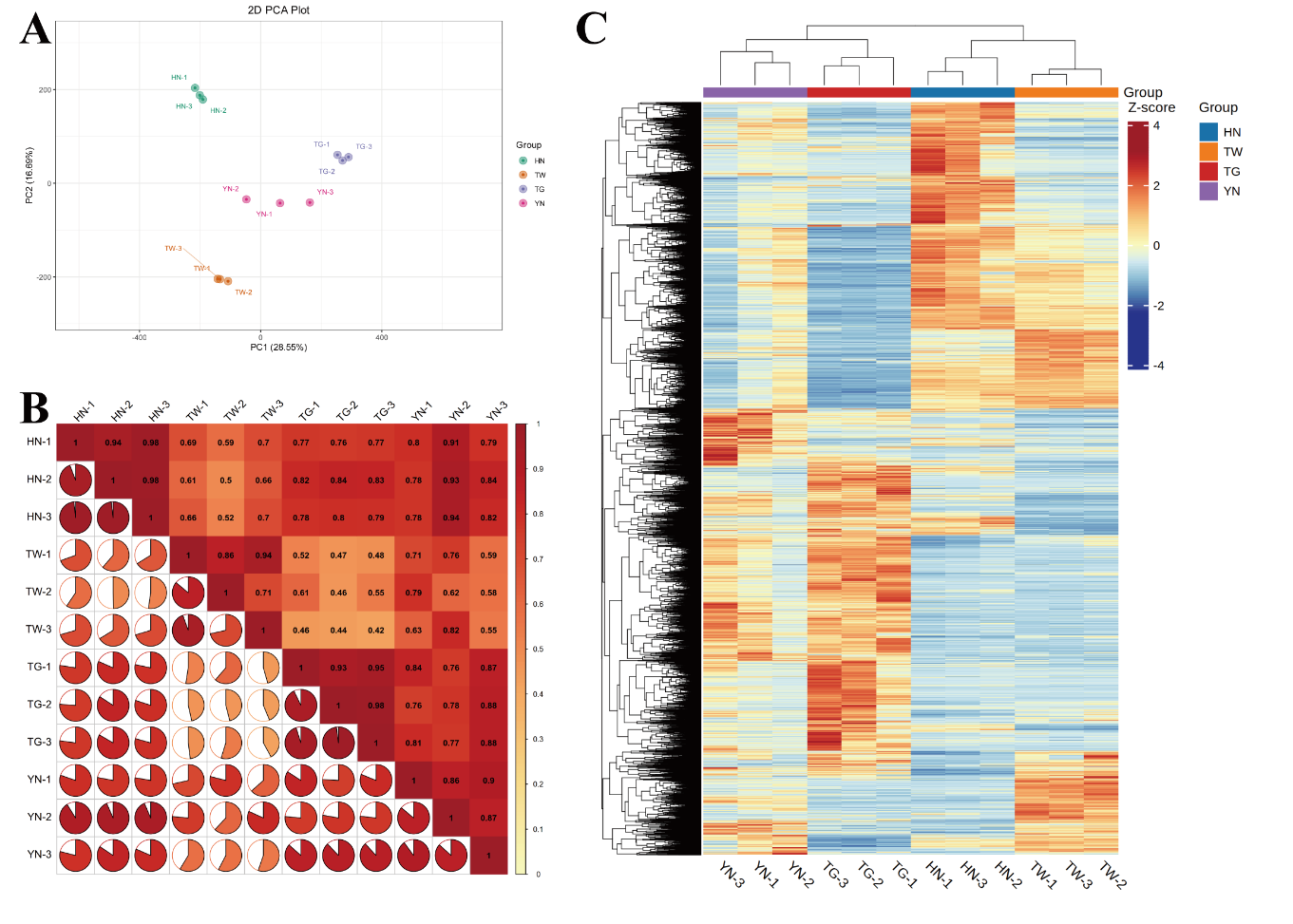


**Supplementary Figure 4.** Transcriptomics profiles of areca nut. (A) PCA score plots of areca nut. (B) Pearson correlation analysis between three biological replicates. (C) Cluster heatmap analysis of DEGs between different varieties.


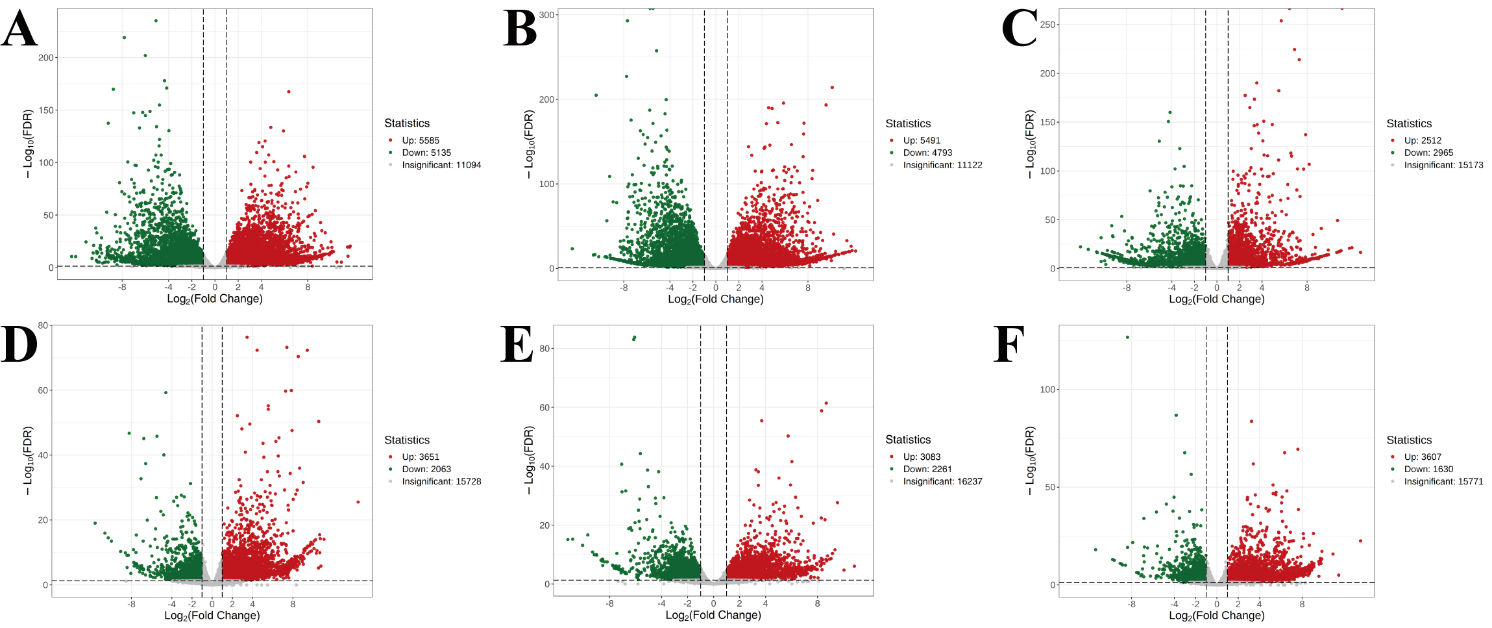


**Supplementary Figure 5.** Volcanic maps of DEGs in different contrasts. (A) TG vs HN; (B) TG vs TW; (C) TW vs HN; (D) YN vs HN; (E) YN vs TG; (F) YN vs TW.


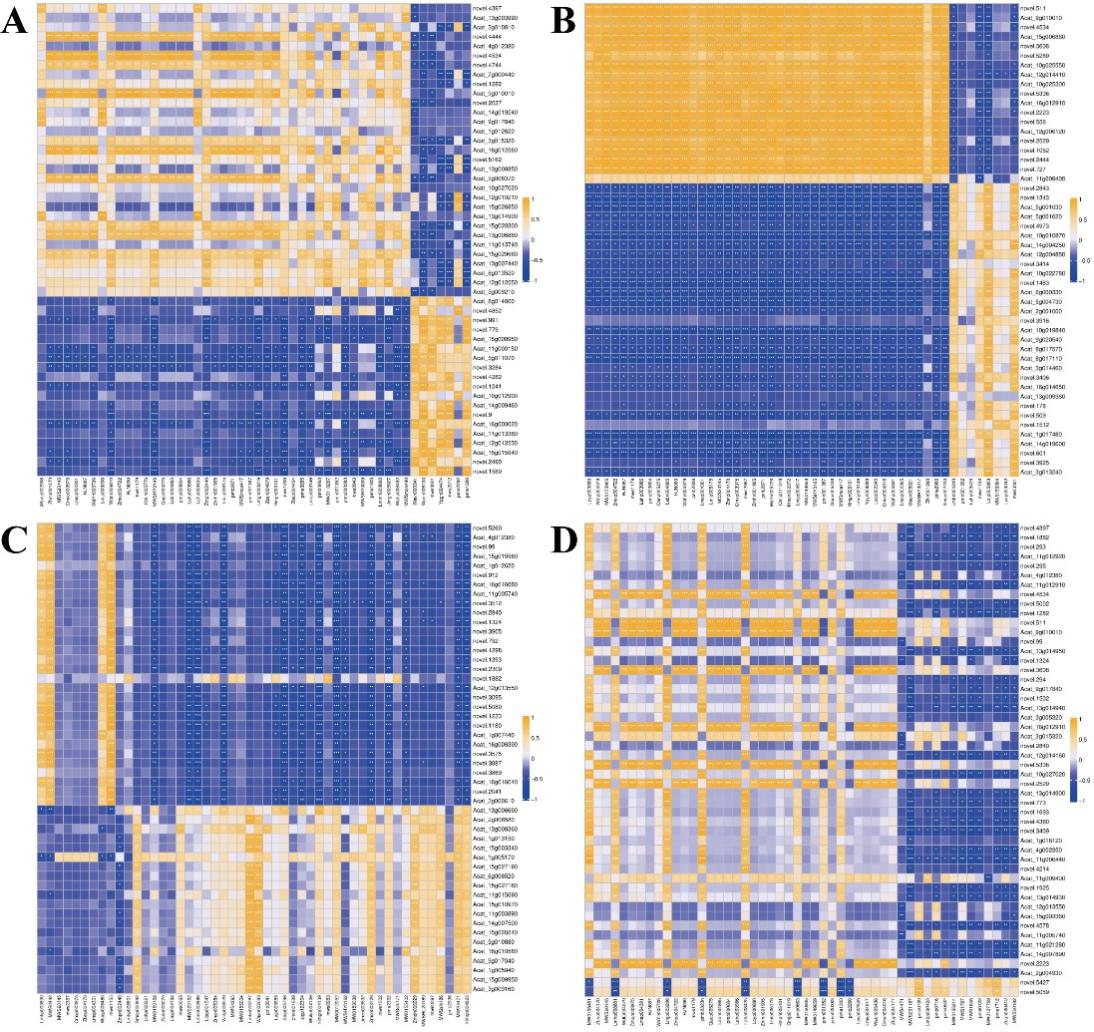


**Supplementary Figure 6.** Correlation coefficient clustering heatmap of the top 50 differentially expressed genes and differentially accumulated metabolites in HN contrast (A), TW contrast (B), TG contrast (C), and YN contrast (D).


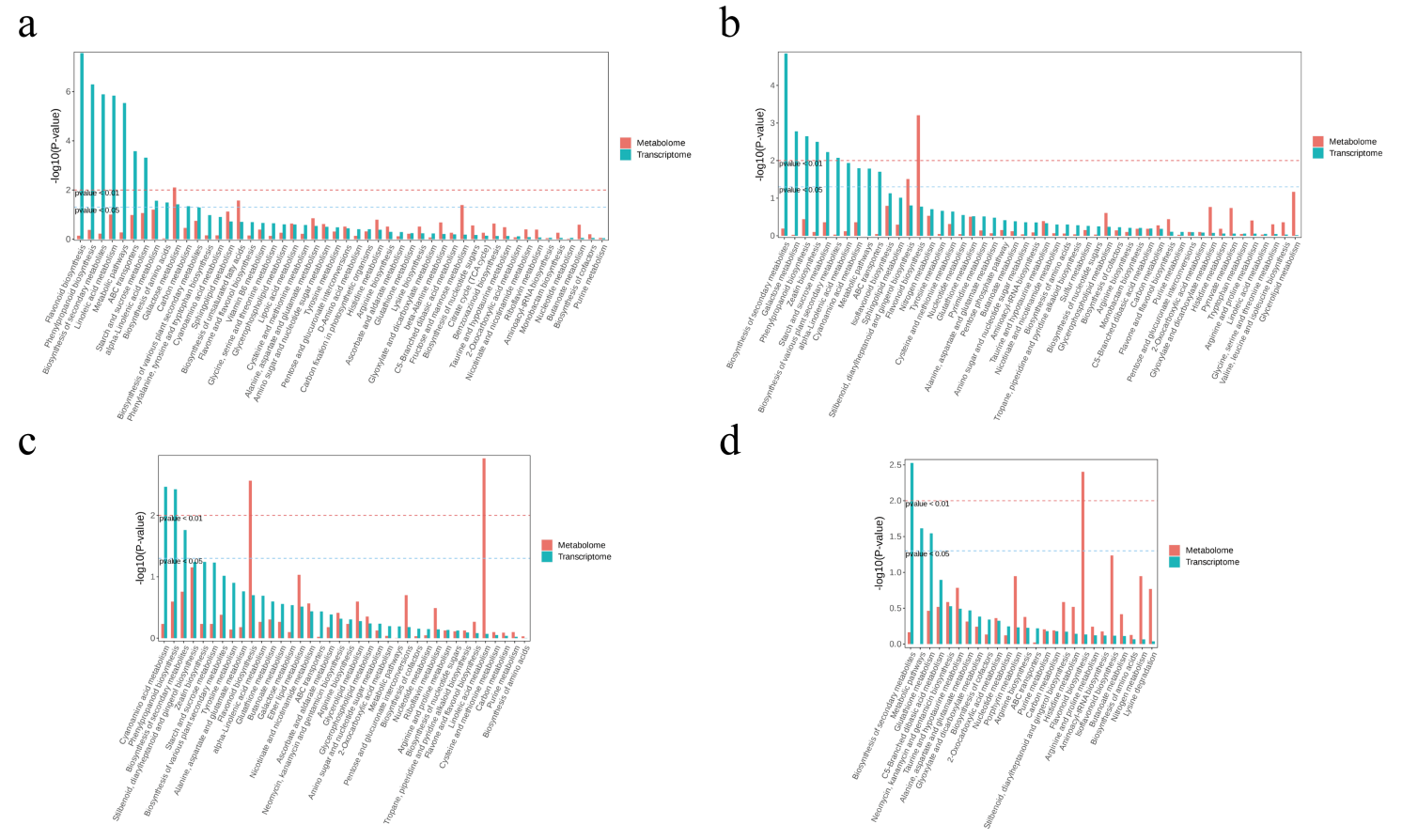


**Supplementary Figure 7.** KEGG co-enrichment analysis of DAMs and DEGs in TG (A), TW (B), HN (C), and YN (D) comparison groups.
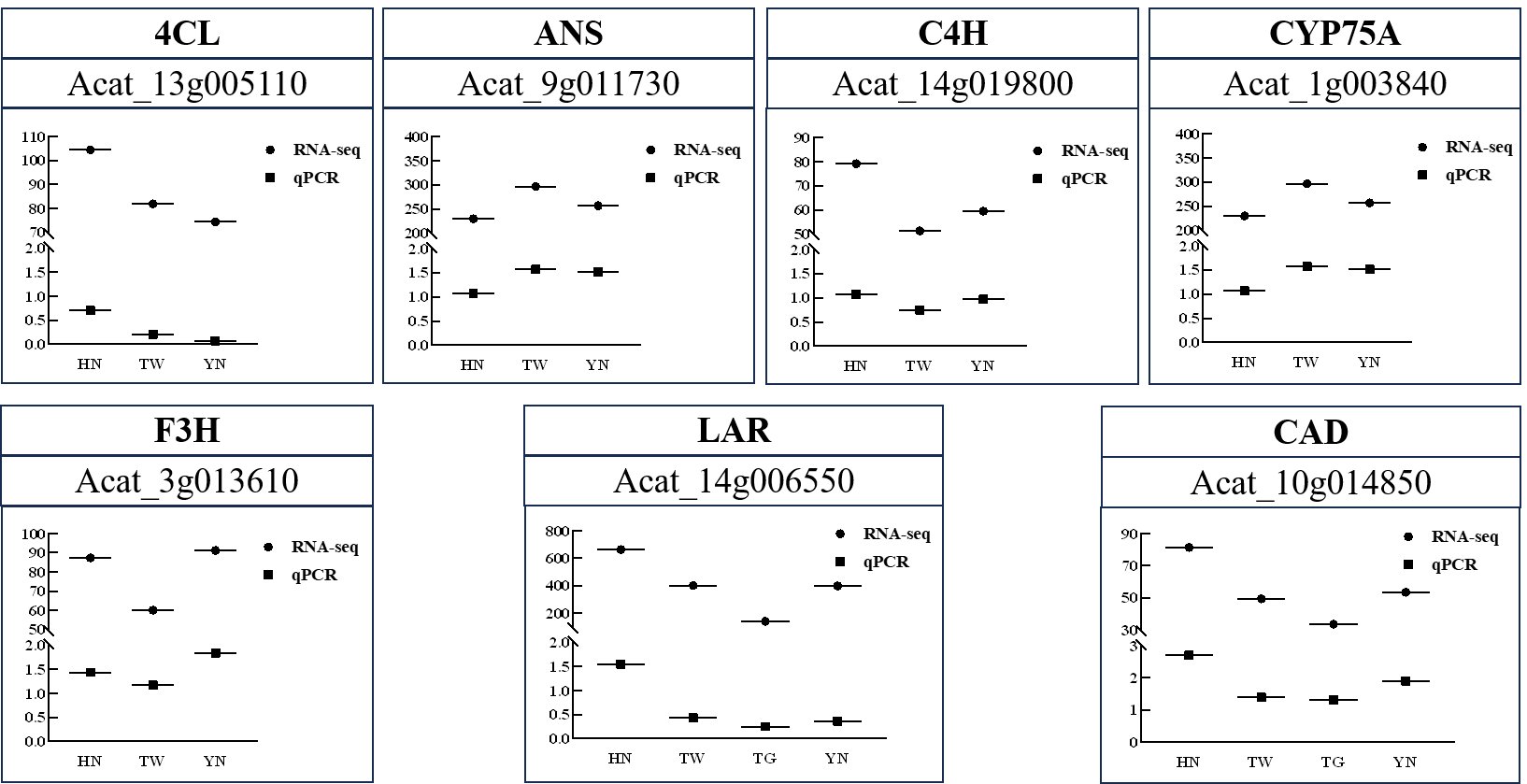


**Supplementary Figure 8.** Comparison of seven DEGs related to phenylpropanoid biosynthesis and flavonoid biosynthesis in HN, TW, TG and YN by RNA-seq and qRT-PCR.

Note: The expression of genes 4CL, ANS, C4H, CYP75A, and F3H in TG is very low, which may cause data inaccuracy. Therefore, TG samples were not verified during qPCR validation.
